# Cdt1 revisited: complex and tight regulation during the cell cycle and consequences of deregulation in mammalian cells

**DOI:** 10.1186/1747-1028-1-22

**Published:** 2006-10-17

**Authors:** Masatoshi Fujita

**Affiliations:** 1 Virology Division, National Cancer Center Research Institute, 5-1-1 Tsukiji, Chuohku, Tokyo 104-0045, Japan

## Abstract

In eukaryotic cells, replication of genomic DNA initiates from multiple replication origins distributed on multiple chromosomes. To ensure that each origin is activated precisely only once during each S phase, a system has evolved which features periodic assembly and disassembly of essential pre-replication complexes (pre-RCs) at replication origins. The pre-RC assembly reaction involves the loading of a presumptive replicative helicase, the MCM2-7 complexes, onto chromatin by the origin recognition complex (ORC) and two essential factors, CDC6 and Cdt1. The eukaryotic cell cycle is driven by the periodic activation and inactivation of cyclin-dependent kinases (Cdks) and assembly of pre-RCs can only occur during the low Cdk activity period from late mitosis through G1 phase, with inappropriate re-assembly suppressed during S, G2, and M phases. It was originally suggested that inhibition of Cdt1 function after S phase in vertebrate cells is due to geminin binding and that Cdt1 hyperfunction resulting from Cdt1-geminin imbalance induces re-replication. However, recent progress has revealed that Cdt1 activity is more strictly regulated by two other mechanisms in addition to geminin: (1) functional and SCF^Skp2^-mediated proteolytic regulation through phosphorylation by Cdks; and (2) replication-coupled proteolysis mediated by the Cullin4-DDB1^Cdt2 ^ubiquitin ligase and PCNA, an eukaryotic sliding clamp stimulating replicative DNA polymerases. The tight regulation implies that Cdt1 control is especially critical for the regulation of DNA replication in mammalian cells. Indeed, Cdt1 overexpression evokes chromosomal damage even without re-replication. Furthermore, deregulated Cdt1 induces chromosomal instability in normal human cells. Since Cdt1 is overexpressed in cancer cells, this could be a new molecular mechanism leading to carcinogenesis. In this review, recent insights into Cdt1 function and regulation in mammalian cells are discussed.

## Background

In eukaryotic cells, genomic DNA is fragmented into multiple chromosomes and DNA replication initiates from multiple replication origins distributed on these. Therefore, the amount of DNA replicated from each origin is relatively short, allowing genome size to expand during the evolution of eukaryotic cells. However, effective operation of the "multiple replication origin" system gives rise to an important problem: *i.e*. multiple replication origins should each be activated precisely only once during each S phase. It is now clear that the "once and only once replication per single cell cycle" is achieved by the periodic assembly and disassembly of essential pre-replication complexes (pre-RCs) at replication origins [1-3]. The pre-RC assembly reaction, known as "licensing", involves the loading of a presumptive replicative helicase, the MCM2-7 complex, onto chromatin by the origin recognition complex (ORC) and two essential factors, CDC6 and Cdt1 [4,5]. The eukaryotic cell cycle is driven by the periodic activation and inactivation of cyclin-dependent kinases (Cdks), pre-RC assembly only occurring in a window of time during the low Cdk period from late mitosis through G1 phase. Thus inappropriate re-assembly is suppressed during S, G2, and M phases.

It was originally suggested that geminin plays a crucial role in suppression of Cdt1 function after S phase in vertebrate cells [6-8] and that Cdt1 hyperfunction resulting from Cdt1-geminin imbalance induces re-replication [9-15]. However, recent progress has revealed that Cdt1 activity is very strictly controlled not only by geminin but also by two other mechanisms after S phase [16-21]. In addition, it has been shown that Cdt1 overexpression evokes chromosomal damage even without inducing re-replication [22]. In this review, recent insights into Cdt1 function and regulation in mammalian cells are summarized and questions that remain to be solved in the future are also discussed.

## Cell cycle regulation of pre-RC assembly in mammalian somatic cells

**Figure 1:**
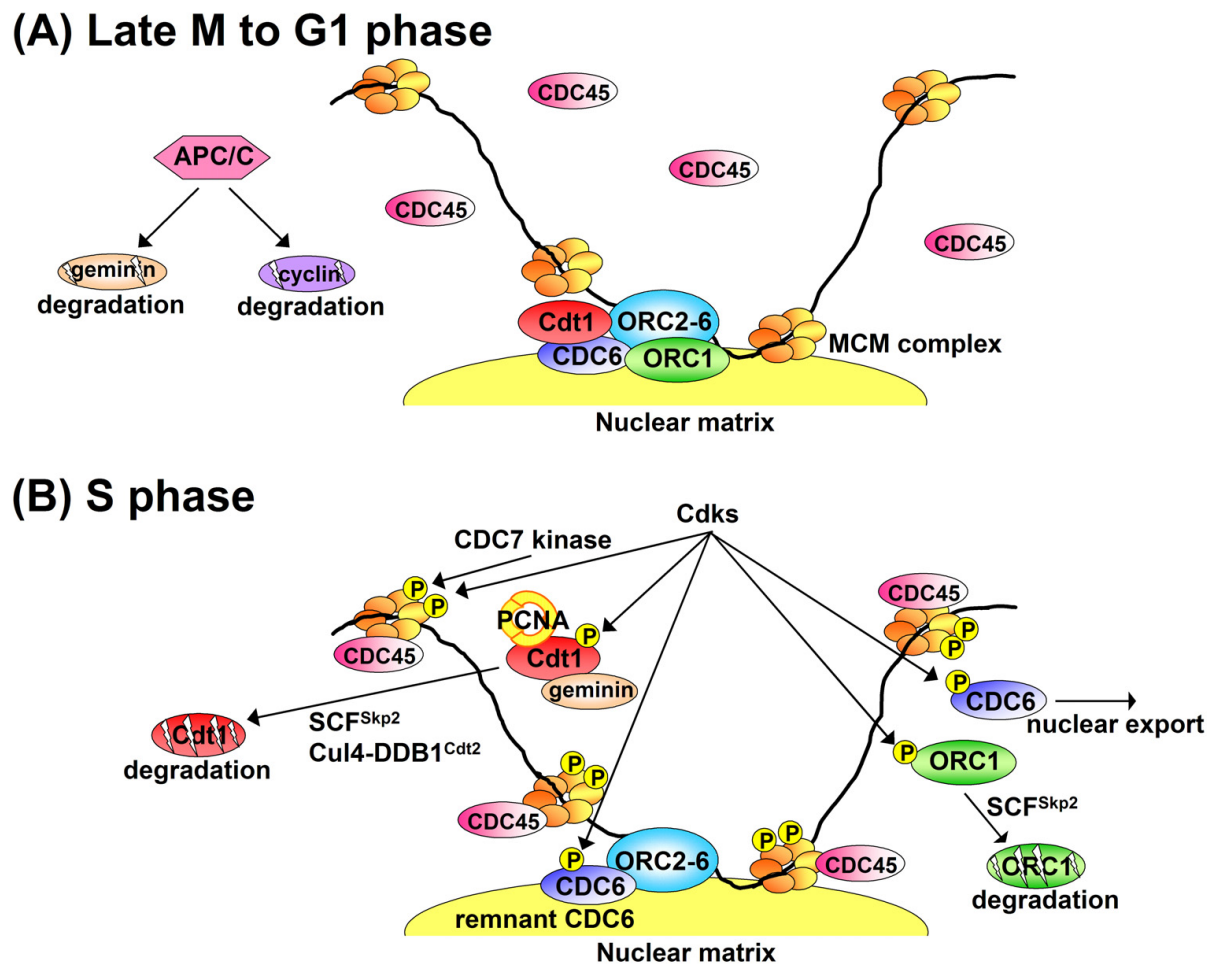
A model for the state of pre-replication chromatin and cell cycle regulation in human cells. (A) During late M to G1 phase when Cdk and geminin activities are suppressed by APC/C ubiquitin ligase, ORC, CDC6 and Cdt1 form the machinery on the nuclear matrix necessary to load MCM2-7 complexes. Multiple MCM complexes are loaded onto chromatin beyond ORC binding sites. (B) When cells enter S phase, CDC45 and some other proteins are recruited around MCM dependent on Cdk and CDC7 kinase activity, unwinding DNA. Then, DNA synthetic proteins are assembled on single-stranded DNA. Activated MCM plays an essential role in DNA replication, probably as a replicative DNA helicase, and is simultaneously displaced from chromatin through an unknown mechanism. The steps after DNA unwinding are omitted in the model shown. After S phase, reloading of dissociated MCM (re-licensing) is suppressed by multiple mechanisms. One is by Cdks, which phosphorylates ORC1 and Cdt1 so that they undergo SCF^Skp2^-mediated proteolysis. Phosphorylation-dependent nuclear export of chromatin-unbound CDC6 could also contribute to inhibition of re-licensing. Cdt1 is further subjected to replication-coupled proteolytic regulation mediated by Cul4-DDB1^Cdt2 ^ubiquitin ligase and PCNA. Geminin also prevents the MCM rebinding by sequestering Cdt1.

In this section, a current model for cell cycle regulation of pre-RC assembly in mammalian cells is summarized (Figure 1). For more detailed and generalized discussion of the licensing reaction and its regulation, see previous review articles [1-3].

Two critical inhibitory factors for the pre-RC assembly are cyclin/Cdks (Cdk1 and Cdk2) and geminin. During late mitosis through the G1 phase, a cell cycle regulatory E3 ubiquitin ligase called the anaphase promoting complex/cyclosome (APC/C) [23,24] is activated and restrains cyclins and geminin by targeting them for proteolysis through polyubiquitination. Thus, pre-RC assembly only occurs during this period (Figure 1A). In mammalian cells, pre-RCs are constructed based on ORC binding to chromatin and the nuclear matrix [25]. Differing from budding yeast ORC, the interaction of mammalian ORC with chromosomal DNA is not simply determined by the primary DNA sequence [1,2,26,27], rather being influenced by high-order chromatin/nuclear structures associated with transcription [28]. It has also been suggested that chromatin regions affixed to the nuclear matrix may more easily access functional ORC because of it association with the matrix [25]. CDC6 and Cdt1 proteins are recruited probably by physical interaction with ORC, and the resultant machinery functions as a loader for the MCM2-7 complex, a presumptive replicative helicase [29,30]. In mammalian cells, multiple MCM complexes appear to be loaded beyond each ORC site [25], which may function as a failsafe mechanism to ensure complete genome duplication [31]. In addition, such a broad distribution of MCM complexes could explain the initiation zones observed in mammalian cells. Finally, it is worthy of note that transcription of ORC1, CDC6, Cdt1 and all MCM subunits is driven by the E2F transcription factor.

At the onset of S phase, Cdk activity is regained following APC/C inactivation, and then pre-RCs initiate replication, accompanied by further assembly of multiple other proteins or protein complexes (Figure 1B) [2]. Firing of pre-RCs is dependent on two kinds of kinases, Cdks and CDC7. Prior to the DNA unwinding step, a series of proteins and protein complexes such as CDC45 and the GINS complex are further loaded to activate MCM helicase [30]. The loading is dependent on both Cdk and CDC7 kinases and once the DNA is unwound, many components of the DNA synthetic machinery are assembled, starting DNA replication [2].

To prevent re-replication, the re-establishment of pre-RC, in other words re-binding of MCM, needs to be suppressed during the S, G2 and M phases of the cell cycle (Figure 1B). Cdks play a central role also in this context [1-3]. In mammalian cells, Cdk1 inactivation in G2 phase results in re-binding of MCM proteins to chromatin and subsequent re-replication [32,33]. Similarly, ablation of cyclin A, but not cyclin B, leads to extensive re-replication in *Drosophila *tissue culture cells [34]. Cdks prevent re-establishment of pre-RC through multiple redundant mechanisms [1-3]. One is by phosphorylation of CDC6. In yeast, this leads to CDC6 degradation [35] while in mammalian cells, nuclear export is the result [36-39]. Such Cdk-dependent nuclear export of CDC6 may at least partly contribute to prevention of re-licensing, but it should be also noted that a significant amount of CDC6 remains bound to chromatin/nuclear matrix through S and G2 phases [39], which may play some role in replication control [40-42]. In human cells, ORC1 is degraded after S phase, presumably depending on phosphorylation by cyclin A/Cdks and binding to SCF^Skp2 ^[25,43]. It has also been shown that the MCM complex is phosphorylated by Cdks [33,44]. In budding yeast, it is necessary to block all three effects of Cdks on ORC, CDC6, and the MCM complex, for induction of re-replication without inhibition of Cdk activity [45]. Also in mammalian cells, deregulation of individual components alone, for example overexpression of ORC1 or CDC6, fails to induce re-replication [9,22,37,38,46], a phenomenon in line with Cdks regulation of multiple pathways.

## Geminin: the first identified component of the Cdt1 inhibitory machinery

**Figure 2:**
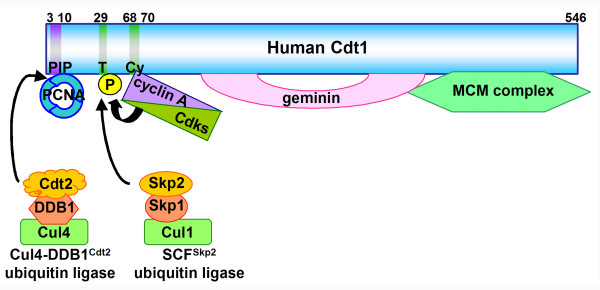
A model for inhibition of Cdt1 function after entry into S phase. Cyclin A/Cdks phosphorylate Cdt1 on threonine 29 depending on cyclin A binding to RXL-type cyclin-binding motif (Cy moif) then SCF^Skp2 ^ubiquitin ligase recognizes phosphorylated Cdt1 and polyubiquitinated Cdt1 is degraded by proteasomes. In addition, Cdk phosphorylation inhibits Cdt1 DNA binding activity. During DNA replication, Cdt1 binds to PCNA on chromatin and Cul4-DDB1^Cdt2 ^ubiquitin ligase recognizes interfaces generated by such Cdt1-PCNA interaction. This mechanism also appears to operate during repair synthesis of damaged DNA although the biological significance remains unclear. After S phase, geminin protein also accumulates, sequestering Cdt1 by direct binding.

Geminin was originally identified as a novel APC/C substrate in *Xenopus*, and found to inhibit pre-RC formation through prevention of the loading of MCM complexes [6]. Subsequent work has shown that geminin inhibits licensing by binding to and inhibiting Cdt1 (Figure 2) [7,8,47]. Since transcription of *geminin *is driven by the E2F transcription factor [48] and geminin protein is an APC/C target, it appears after cells enter S phase and is destroyed during exit from mitosis to allow pre-RC formation [6-8].

At least in certain cancer-derived cell lines, depletion of geminin by siRNA induces some re-replication [10,11]. Therefore, it is clear that geminin is a crucial factor for preventing re-licensing and subsequent re-replication in mammalian cells. However, in other cell types, geminin depletion does not evoke overt re-replication [49]. In *Xenopus *egg extracts, immunodepletion of geminin does not induce extensive re-replication [12-15]. Also in *Drosophila *tissue culture cells, in contrast to cyclin A depletion, geminin depletion results in only partial re-replication [34]. Therefore, both Cdks and geminin would appear to be indispensable to securely suppress re-replication under any circumstances. Cdk-independent inhibition of pre-RC assembly by geminin would be favored in some situations; *e.g*. even when Cdk activity is down-regulated by the checkpoint mechanism in cells undergoing DNA damage, re-formation of pre-RC can be prevented by geminin.

Recently, new functions of geminin, independent of Cdt1 binding and related to transcriptional regulation, have been successively uncovered [50-52]. Considering that geminin homologues are not found in yeasts, it may had evolved in contexts other than regulation of DNA replication and thereafter have become adapted for roles in Cdt1 regulation.

## Cdt1 phosphorylation by Cdks: a second component of the inhibitory machinery

Although Cdt1 function is suppressed by geminin after S phase, Cdt1 might also be regulated by Cdks. Indeed, it was found that Cdt1 is phosphorylated by cyclin A-dependent kinases (cyclin A/Cdk1 and cyclin A/Cdk2) dependent on the RXL-type cyclin-binding motif (Cy motif) [16,17]. Cdk phosphorylation results in Cdt1 binding to the F-box protein Skp2, a component of the SCF (Skp1-Cullin1-F-box protein) ubiquitin ligase complex, and its subsequent degradation [16,17,53,54]. Thus, Cdks regulate Cdt1 via phosphorylation-dependent proteolysis (Figure 2). However, a Cdt1 Cy mutant, which is refractory to Cdk phosphorylation and SCF^Skp2 ^recognition, showed only partial resistance to degradation during S phase in Rat-1 cells [16]. Also in HeLa cells, it has been observed that Cdt1 mutants deficient in Cdk phosphorylation and subsequent SCF^Skp2 ^binding are still degraded with comparable efficiency to the wild type [54]. These findings clearly show that there is a separate mechanism(s) that targets Cdt1 for proteolysis during S phase (see below). Cdk phosphorylation also down-regulates Cdt1 via a mechanism distinct from the proteolysis, impairing its *in vitro *DNA binding activity [16]. In cells arrested around G2/M phase, levels of Cdt1 protein are not lowered but it remains detached from chromatin. When Cdk1 is inactivated in such cells, Cdt1 is dephosphorylated and rebinds to chromatin [16]. Cdk phosphorylation-mediated inhibition of Cdt1 DNA binding activity provides one possible explanation for such phenomenon.

Unscheduled Cdt1 hyperfunction induces re-replication [9] and chromosomal damage without re-replication [22]. In Rat-1 cells, the Cdt1 Cy mutant induces stronger chromosomal damage than the wild type [22]. Also in HeLa cells, Cdt1 mutants deficient in Cdk phosphorylation exhibit more extensive re-replication, despite their efficient degradation [21,54]. These findings support an importance for Cdt1 regulation by Cdks, especially in a proteolysis-independent manner such as inhibition of Cdt1 DNA binding activity. Cdk-mediated regulation of Cdt1 has subsequently also been noted in *Drosophila *[55].

In mammalian cells, Cdk1 inactivation in G2 phase promotes inappropriate re-licensing [33]. The question therefore arises why geminin cannot inhibit re-licensing under this circumstance. Are interactions between Cdt1 and geminin directly affected by Cdks? This may not be the case [16]. Geminin is a substrate of APC/C^Cdh1 ^ubiquitin ligase and Cdks restrains this ligase by phosphorylating Cdh1 subunit [23,24]. Therefore, Cdk inhibition could simultaneously induce geminin inactivation by APC/C. In this regard, it is notable that APC/C-mediated inhibition of geminin does not necessarily require degradation by a proteasome and ubiquitination itself may be sufficient [56]. A more simple explanation could be that only geminin binding is insufficient to inhibit Cdt1 when it escapes from degradation.

## One more piece of Cdt1 inhibitory machinery: replication-coupled degradation mediated by the Cullin4 (Cul4)-based ubiquitin ligase and proliferating cell nuclear antigen (PCNA)

Recently, Cul4-based ubiquitin ligase- and PCNA-mediated proteolysis has emerged as a third mechanism regulating Cdt1 during the S phase (Figure 2). Four groups have reached the same conclusion, as successively reported in the beginning of this year [18-21].

Cul4 is a member of the Cullin ubiquitin ligase family [57,58], together with the SCF constructed based on Cul1 [23,24,59]. Involvement of Cul4 in regulation of Cdt1 function was first suggested in *C. elegans*, in which ablation of Cul4 by RNAi induces re-replication which is in turn suppressed by removal of one genome copy of *Cdt1 *[60]. The level of *C. elegans *Cdt1 protein is decreased as cells enter S phase, and this decrease disappears with Cul4 depletion. Thus, the Cul4 ubiquitin ligase appears to regulate Cdt1 stability during S phase, albeit by a mechanism which remains to be elucidated. Subsequently, it was found that in human cells, Cdt1 is rapidly targeted for proteasome-mediated degradation after DNA damage by UV irradiation and that this proteolysis involves ubiquitination of Cdt1 by Cul4-DDB1 ligase [61,62]. It has been shown that Cul4-DDB1 ubiquitin ligase immunopurified from cells can catalyze Cdt1 polyubiquitination *in vitro *and that DDB1 can directly bind to Cdt1 [61,62]. However, it remains unclear how Cdt1 is specifically recognized by Cul4-DDB1 ligase only when chromatin is damaged. Furthermore, the biological significance of Cdt1 degradation after chromatin damage has also been somewhat vague. Once MCM complexes are loaded onto chromatin, ORC, CDC6, and Cdt1 appear to be no longer required for the initiation reaction. Therefore, Cdt1 removal after DNA damage might not be expected to contribute to blocking S phase entry.

Recent progress has resolved this question. Cdt1 has a PCNA-interaction protein motif (PIP motif; QxxI/L/VxxFF) [63,64] in the N-terminus, which is conserved from *C. elegans *to mammalian cells [18-21]. As mentioned above, mutations that abrogate Skp2 binding cannot completely stabilize human Cdt1 during S phase. Interestingly, it was shown that additional mutations in the PIP motif does block S phase degradation [19,21]. Silencing of Cul4 or DDB1 by siRNA also stabilizes mutant Cdt1 deficient in Skp2 binding [19,21]. Thus, proteolytic regulation of human Cdt1 during S phase is carried out by two redundant pathways, involving SCF^Skp2^- and Cul4-DDB1-mediated ubiquitination. Indeed, co-inhibition of both pathways by siRNA can stabilize wild type human Cdt1 in S phase [21]. The finding that the PIP motif is required for Cul4-DDB1-mediated proteolysis indicates a possible involvement of PCNA. In fact, Cdt1 binds to PCNA via the PIP motif and silencing of PCNA by siRNA as well as the PIP mutation can stabilize the mutant Cdt1 deficient in Skp2 binding during the S phase [19,21]. Essentially the same results have been also obtained with an *in vitro *DNA replication system using *Xenopus *egg extracts although the Cdk-SCF^Skp2 ^system does not seem to operate in this case [18]. Considering that ablation of Cul4 induces overt re-replication in *C. elegans*, Cul4-mediated degradation of Cdt1 may be of prime importance in this organism although it remains possible that Cul4 is also involved in other systems associated with replication control (see below). Interestingly, Cdt1 proteolytic regulation involving PCNA and Cul4 is also observed in fission yeast [20]. We can conclude that a strict regulation system has evolved to control Cdt1 (Figure 2).

PCNA, a eukaryotic sliding clamp, is present in the nuclei throughout the cell cycle and loaded onto the primer ends synthesized on unwound DNA during S phase. Then, it recruits replicative DNA polymerases and stimulates their activity [63,64]. PCNA also recruits many other PCNA-binding proteins which regulate replication-associated processes [63,64]. Although detailed mechanisms remain to be clarified, a line of evidence indicates that only chromatin-loaded PCNA can trigger Cdt1 ubiquitination by Cul4-DDB1, ensuring a timely, replication-coupled degradation of Cdt1 to prevent re-licensing [18,19,21]. Very importantly, a prokaryote, *E. coli *also employs a similar strategy to prevent re-initiation of DNA replication, where an initiation factor, DnaA is inactivated via a prokaryotic sliding clamp, the β clamp [65,66]. Thus, DNA replication-coupled, sliding clamp-mediated inactivation of initiation factors appears to be a universal system to prevent re-replication and maintain genome stability. PCNA is also loaded onto chromatin during repair synthesis of damaged DNA that operates outside of S phase. As expected from this, DNA damage-induced, Cul4-DDB1 ligase-mediated Cdt1 degradation also depends on PCNA-Cdt1 interaction [19-21]. The DNA damage-induced Cdt1 degradation might be a "by-product" of machinery for replication-coupled degradation. An intriguing question is whether DNA damage-induced Cdt1 degradation indeed contributes to genome stability.

Several other important points also need to be resolved. Cul4-DDB1 ubiquitin ligase may recognize an interface generated by Cdt1-PCNA interaction, but it is unclear how this ligase only recognizes Cdt1-PCNA complexes formed on chromatin. One simple explanation is that functional Cul4-DDB1 could be always tethered to chromatin. Since there are many PCNA-binding proteins, it is of interest why Cdt1 (and possibly some other targets) is ubiquitinated by Cul4 and brought to proteolysis while others are not. In this context, it should be noted that the *Xenopus *p27^Xic1 ^Cdk inhibitor is also targeted to degradation in a PCNA-dependent manner, although involvement of Cul4 has not been examined [67]. Presumably, Cul4-DDB1 ligase recognizes specific interfaces generated between PCNA and target proteins that should be ubiquitinated. It is conceivable that in addition to the PIP motif, some specific amino acid residues are also required for recognition by Cul4-DDB1 ligase, identification of which is a worthy research challenge. By analogy with SCF ubiquitin ligase, Cul4 is equivalent to Cul1 and DDB1 may be equivalent to Skp1 (Figure 2) [57-59]. In the SCF ubiquitin ligase, each of many F-box proteins that bind to Skp1 acts as a specific substrate-recognition subunit and in the case of Cdt1, it is Skp2 [16,17]. So, what are the proteins playing roles in Cdt1 recognition by Cul4-DDB1 ligase? During drafting of this review, a WD40-repeat-containing protein, Cdt2 was identified as a candidate substrate receptor for the Cdt1-PCNA interface, although direct proof is still lacking [68,69]. It should be noted that Cdt2 was originally identified in a same screening with Cdt1 in fission yeast but that they are structurally unrelated [70]. Finally, ubiquitination by Cul4-based ligase has been also implicated in regulation of other important chromatin proteins, including DNA repair-related factors, core histones and p53 [58,71]. Therefore, it may be necessary to consider cross-integration of these different chromatin transactions by Cul4 ubiquitin ligase.

## Why is Cdt1 regulated so strictly by multiple mechanisms and what occurs when the strict regulation is perturbed: to be or not to be re-replicated?

The fact that Cdt1 activity is controlled by multiple mechanisms in human cells suggests that its deregulation may induce more deleterious insult than with other pre-RC components such as ORC1 and CDC6. Indeed, it is reported that overexpression of Cdt1 can induce overt re-replication in cancer-derived cell lines, with activation of ATM/ATR checkpoint pathways [9]. This contrasts to the fact that overexpression of ORC1 or CDC6 has no or only marginal effects on cell cycle progression [9,22,37,38,46]. As discussed above, Cdks utilize multiple mechanisms to prevent re-replication (Figure 1B). For example, it has been reported that ORC1 protein is degraded after entry into S phase in several cancer-derived cell lines [25,43]. This is also the case in all human cell lines we have tested (including non-transformed cell lines), without exception (own unpublished data). Naturally, Cdt1 cannot compensate for ORC1 function. So, why can overexpression of Cdt1 alone induce overt re-replication? The fact that Cdt1-induced re-replication has been observed in cancer-derived cell lines could provide an answer. Cancer cells constitutively overexpress replication initiation factors such as ORC1, CDC6, Cdt1, and MCM [22], so that ORC1 degradation in S phase may be insufficient to fully suppress its function. Consistent with this idea, we have observed that cell growth is severely inhibited in normal human fibroblasts when ORC1 protein level is reduced by ~80% with shRNA expression while it is not in cancer-derived HeLa cells (own unpublished data). Cdt1-induced re-replication is also observed in the *Xenopus *egg extract system [12-15], a system that may be representative of rapid early embryonic cell cycling in vertebrates. The cause could be large amounts of maternal initiation proteins in the eggs. It has been also suggested that ORC function is not inhibited during S phase in this system [72]. Under physiological conditions, strict control of Cdt1 by multiple pathways may be particularly crucial in situations characterized by rapid cell cycling such as in the early embryo [73]. It has been proposed that induction of re-replication by Cdt1 overexpression in cancer cells is suppressed by p53 activated by ATM/ATR pathways [9]. On the other hand, re-replication upon geminin depletion in cancer cells is observed regardless of the p53 status [10,11]. The reason for this apparent difference is not clear at present.

In non-transformed cultured cells, Cdt1 overexpression at pathophysiological levels appears not to induce re-replication but nevertheless leads to chromosomal damage, as revealed by activation of the ATM-Chk2 DNA damage checkpoint pathway [22]. Furthermore, deregulation of Cdt1 causes such damage even in quiescent cells and is associated with chromosomal instability in normal human cells [22]. Together, these findings demonstrate that the strict regulation of Cdt1 is also important for the normal somatic cell cycle. The mechanisms by which Cdt1 overexpression negatively impacts on chromatin remain to be clarified. One possibility is that DNA double-strand breaks are involved, although their direct detection has so far not been reported. Another possibility is that the presence of Cdt1 in the quiescent state or after S phase inappropriately changes chromatin architecture, either directly or indirectly through recruiting other protein(s), activating ATM [74]. As components of the MCM complex-loading machinery, ORC and CDC6 proteins act using their ATPase activity as does replication factor C, a loader for PCNA [2]. However, it remains unclear how Cdt1 acts during MCM loading. Alteration of the chromatin structure by Cdt1 could be related to its physiological role in MCM loading, an intriguing possibility that should be tested in future.

There is evidence that silencing of geminin induces re-replication, suggested to be attributable to Cdt1 deregulation [10,11]. However, it should be noted that cancer cells have been used in most of the reported studies and geminin has roles other than in Cdt1 inhibition, for example in transcriptional regulation [50-52]. Similarly with the re-replication due to inhibition of Cul4-DDB1^Cdt2 ^by RNAi [60,68,75], it should be considered that the ligase may act on target proteins other than Cdt1, including Cdk inhibitors as discussed above. In mammalian cells, PIP motif-mutated Cdt1 is still degraded in S phase and shows reduced ability to induce re-replication compared with the wild type [21,54]. On the other hand, in the *Xenopus *egg extract system, PIP motif-mutated Cdt1 is stabilized and induces more re-replication than the wild type [18]. This could represent a difference in cell cycle regulation between embryonic and somatic cells.

## Is Cdt1 an oncogene?

It has been reported that Cdt1 overexpression endows murine NIH3T3 cells with the capacity to form tumors in nude mice [76]. However, in Rat-1, another cell line commonly used to estimate transformation potency of classical oncogenes, no transformed phenotype was observed with stable overexpression of Cdt1 [22]. This difference might simply be attributable to cell type-specific responses. However, there is abundant evidence that deregulation of Cdt1 impacts on cells by inducing re-replication, chromosomal damage, and genomic instability [9,22]. Thus, I prefer the explanation that deregulated Cdt1 does not lead to acute oncogenic transformation, but rather to chronic chromosomal damage and instability that eventually results in transformation. In fact, Cdt1 overexpression in transgenic thymocytes by itself does not lead to tumor formation but modulates and enhances tumor formation induced by p53 deficiency [77].

Importantly, Cdt1 protein is actually overexpressed in human cancer cells [78,79,22]. *Cdt1 *transcription is driven by E2F transcription factor [48], which is often deregulated in cancer cells, for example by RB pathway disturbance. Thus, Cdt1 deregulation would be a novel molecular mechanism leading to carcinogenesis.

## Conclusion

In mammalian cells, Cdt1 is a very central player in regulation of DNA replication. During the cell cycle, strict control is exerted by geminin, by Cdk phosphorylation and subsequent SCF^Skp2^-mediated proteolysis, and by replication-coupled proteolysis involving Cul4-DDB1^Cdt2 ^and PCNA. Details of these multiple regulations have only recently been uncovered and many questions remain, including how Cdt2 specifically recognizes interfaces between Cdt1 and PCNA on chromatin and how Cul4-DDB1 ubiquitin ligase coordinates several related chromatin transactions. As expected from the strict regulation, deregulation of Cdt1 is a deleterious insult, leading to re-replication and/or chromosomal damage. The induced chromosomal instability may eventually lead to carcinogenesis and Cdt1 overexpression is in fact often observed in human cancers. The mechanisms by which Cdt1 can damage chromatin without inducing re-replication are unclear at present and this is an interesting research challenge. Elucidation could provide valuable information regarding the essential question of how Cdt1 functions in MCM loading reaction.

## Competing interests

The author(s) declare that they have no competing interests.

## Authors' contributions

MF wrote the entire manuscript.
